# An Exome-Wide Sequencing Study of the GOLDN Cohort Reveals Novel Associations of Coding Variants and Fasting Plasma Lipids

**DOI:** 10.3389/fgene.2019.00158

**Published:** 2019-02-26

**Authors:** Xin Geng, Marguerite R. Irvin, Bertha Hidalgo, Stella Aslibekyan, Vinodh Srinivasasainagendra, Ping An, Alexis C. Frazier-Wood, Hemant K. Tiwari, Tushar Dave, Kathleen Ryan, Jose M. Ordovas, Robert J. Straka, Mary F. Feitosa, Paul N. Hopkins, Ingrid Borecki, Michael A. Province, Braxton D. Mitchell, Donna K. Arnett, Degui Zhi

**Affiliations:** ^1^School of Biomedical Informatics, The University of Texas Health Science Center at Houston, Houston, TX, United States; ^2^BGI-Shenzhen, Shenzhen, China; ^3^Department of Epidemiology, The University of Alabama at Birmingham, Birmingham, AL, United States; ^4^Department of Biostatistics, The University of Alabama at Birmingham, Birmingham, AL, United States; ^5^Division of Statistical Genomics, Department of Genetics, Washington University School of Medicine, St. Louis, MO, United States; ^6^USDA/ARS Children’s Nutrition Research Center, Baylor College of Medicine, Houston, TX, United States; ^7^Division of Endocrinology, Diabetes and Nutrition, Department of Medicine, University of Maryland School of Medicine, Baltimore, MD, United States; ^8^Nutrition and Genomics Laboratory, Jean Mayer United States Department of Agriculture Human Nutrition Research Center on Aging, Tufts University, Boston, MA, United States; ^9^IMDEA Alimentación, Madrid, Spain; ^10^Centro Nacional de Investigaciones Cardiovasculares, Madrid, Spain; ^11^Department of Experimental and Clinical Pharmacology, University of Minnesota, Minneapolis, MN, United States; ^12^Division of Cardiovascular Medicine, University of Utah, Salt Lake City, UT, United States; ^13^Genetic Analysis Center, Department of Biostatistics, University of Washington, Seattle, WA, United States; ^14^College of Public Health, University of Kentucky, Lexington, KY, United States; ^15^School of Public Health, The University of Texas Health Science Center at Houston, Houston, TX, United States

**Keywords:** whole exome sequencing, rare variant, HDL, LDL, triglyceride, cholesterol, genetics, epidemiology

## Abstract

**Background:** Associations of both common and rare genetic variants with fasting blood lipids have been extensively studied. However, most of the rare coding variants associated with lipids are population-specific, and exploration of genetic data from diverse population samples may enhance the identification of novel associations with rare variants.

**Results:** We searched for novel coding genetic variants associated with fasting lipid levels in 894 samples from the Genetics of Lipid Lowering Drugs and Diet Network (GOLDN) with exome-wide sequencing-based genotype data. In single variant tests, one variant (rs11171663 in *ITGA7*) was associated with fasting triglyceride levels (*P* = 7.66E-08), explaining approximately 3.2% of the total trait variance. In gene-based tests, we found statistically significant associations between *ITGA7* (*P* = 1.77E-07) and *SLCO2A1* (*P* = 7.18E-07) and triglycerides, as well as between *POT1* (*P* = 3.00E-07) and low-density lipoprotein cholesterol. In another independent replication cohort consisting of 3,183 African American samples from Hypertension Genetic Epidemiology Network (HyperGEN) and the Genetic Epidemiology Network of Arteriopathy (GENOA), the top genes achieved *P*-values of 0.04 (*ITGA7*), 0.08 (*SLCO2A1*), and 0.02 (*POT1*). In GOLDN, gene transcript levels of *ITGA7* and *SLCO2A1* were associated with fasting triglycerides (*P* = 0.07 and *P* = 0.02), highlighting functional relevance of our findings.

**Conclusion:** In this study, we present preliminary evidence of novel rare variant determinants of fasting lipids, and reveal potential underlying molecular mechanisms. Moreover, these results were replicated in an independent cohort. Our findings may inform novel biomarkers of disease risk and treatment targets.

## Introduction

The understanding of the biology behind lipid-metabolism has increased exponentially in the past two decades, enabled by the vast interrogation of the human genome. To date, genome-wide association studies have identified over 170 candidate single-nucleotide variants (SNVs), with many residing in common regions of the genome (Teslovich et al., 2010; Do et al., 2013). However, two major limitations exist in the current exploration for causal loci associated with lipid metabolism: (1) Identification of SNVs in non-coding regions or SNVs in large regions that span several candidate genes; and (2) missing detection of candidate genes altogether, particularly if population-specific (Assimes and Quertermous, 2014). Exome-wide association studies naturally expand on findings from genome-wide association studies through their exploration of the functional region of the genome (Majewski et al., 2011).

Exome-wide association studies have been extensively used to dissect the genetic architecture of complex diseases and quantitative traits (Lee et al., 2014). Exonic variants, particularly loss-of-function variants, tend to show the most dramatic effect sizes, yielding the greatest power for detection. Recent evidence on lipid traits provides support that rare variants can be ancestry-specific (Lu et al., 2017). Therefore, examining exonic variants across diverse ancestry groups likely augments the identification of novel loci.

The Genetics of Lipid Lowering Drugs and Diet Network (GOLDN) cohort, recruited from families of European ancestry residing in the United States, provides an opportunity to study the effects of rare genetic variants on clinically measured lipid levels. Prior studies in GOLDN have identified common variants controlling lipid fasting level (An et al., 2014). To augment the discoveries which were limited to common variants and identify additional rare functional loci contributing to variation in fasting plasma lipid level, we performed an exome-wide sequencing study in 894 GOLDN participants to identify novel associations of coding variants and fasting lipid traits. To further explore population-specific effects, we also performed replication analyses in two diverse external validation cohorts: (1) the Heredity and Phenotype Intervention (HAPI) Heart Study, composed of Old Order Amish individuals; and (2) the Hypertension Genetic Epidemiology Network (HyperGEN) and the Genetic Epidemiology Network of Arteriopathy (GENOA), which both recruited African Americans. Finally, we interrogated the functional relevance of our significant findings using DNA methylation and RNA-Seq data available in GOLDN.

## Materials and Methods

### Study Populations

Genetics of Lipid Lowering Drugs and Diet Network (clinicaltrials.gov-NCT00083369) recruited and sequenced 894 participants from 186 families of European ancestry at two centers (Minneapolis, MN, United States and Salt Lake City, UT, United States) to characterize genetic and epigenetic determinants of lipid levels (Wojczynski et al., 2015). The population size indicates we have statistical power ranging from 0.5 for h^2^_locus_ = 0.02 to 1.00 for h^2^_locus_ = 0.05 or above in the single variant test, and from 0.8 for h^2^_locus_ = 0.02 to 1.00 for h^2^_locus_ = 0.05 or above in the gene-based test for SNVs with MAF < 0.05. The participants were healthy without diabetes or cardiovascular disease, and they were asked to discontinue any lipid-lowering agents (pharmaceuticals or nutraceuticals) for at least 4 weeks prior to the initial visit. Demographic and clinical characteristics of the study participants are listed in Table 1.

**Table 1:** Demographic and clinical characteristics of samples in GOLDN, HyperGEN and GENOA, and HAPI Heart Study.

	GOLDN	HyperGEN and GENOA	HAPI Heart Study
Sex	Male: 435	Male: 488	Male: 404
	Female: 459	Female: 881	Female: 366
Age	50.2 ± 6.1	48.9 ± 11.26	43.50 ± 13.90
Recruiting	Minneapolis, MN: 457	Alabama: 1042	–
Center	Salt Lake City, UT: 437	North Carolina: 327	
BMI (kg/m^2^)	28.5 ± 5.6	32.05 ± 7.6	26.62 ± 4.46
LDL (mg/dL)	122.77 ± 31.88	122.06 ± 36.87	–
HDL (mg/dL)	46.73 ± 13.06	54.09 ± 15.84	–
TG (mg/dL)	139.34 ± 97.63	109.26 ± 74.03	68.56 ± 41.37


We sought to replicate the significant association in two studies. The effect directions of the associated variants were also compared in different ethnic populations. The first study is HAPI Heart Study consisting of 770 Old Order Amish participants. The triglyceride (TG) levels were relatively low in the physically active Amish population (Mitchell et al., 2008; Table 1). It was allowed to use HAPI Heart Study for replication of the TG findings, not the LDL-C for *POT1*. The second study, which consisted of 3,183 African Americans from pooled samples of HyperGEN and GENOA, was used to replicate high-density lipoprotein cholesterol (HDL-C), low-density lipoprotein cholesterol (LDL-C), and TG findings. In the replication test, only the top genes found in GOLDN were analyzed. All the exonic variants within these top genes in the replication populations were included in the gene-based test.

### Genotype, Phenotype, and Statistical Analysis

The sequencing, genotyping, and quality control procedures were described in a prior study (Geng et al., 2018). Briefly, genomic DNA from peripheral blood nucleated cells was extracted using QIAmp 96 DNA Blood Kits (Qiagen, Hilden, Germany). After Illumina paired end small fragment libraries were constructed, they were run on a HiSeq 2000 V3 2 bp × 101 bp sequencing run. Illumina sequencing data in FASTQ format were aligned to the GRCh37-lite reference sequence using BWA (Li and Durbin, 2010) version 0.5.9. SNVs were called using the Atlas-SNP2 first at the Subject-level and then combined (Challis et al., 2012). Only biallelic mutations were kept after filtered using VCFtools (Danecek et al., 2011). For the genotype level QC, genotypes with read depth less than 20 or genotyping quality less than 30 were excluded. For the variant level QC, the mutations were filtered out if their missing rate exceeded 5%. The project-level VCF was further annotated using ANNOVAR (Wang K. et al., 2010) according to hg19 genome assembly/dbSNP version 138. Four classes of functional variants (splicing, non-synonymous, stop-loss, and stop-gain) on chromosomes 1–22 were used for association tests (Lange et al., 2014). We required that >70% of target bases were covered at >20×; samples below that threshold received additional (top-up) sequencing. To confirm sample purity and identity, we compared high-density SNVarray genotypes (Aslibekyan et al., 2012) (Illumina Omni Express) to the SNV calls, and each sample achieved >90% genotype concordance.

HDL-C, LDL-C, and TG were measured in this study in the fasting state as previously reported (Liu et al., 2008). All the lipid values were natural log transformed to achieve normality of residuals. Genetic associations were assessed using linear mixed models using RAREMETALWORKER and RAREMETAL (version 4.13.6). All the associations were adjusted for sex, age (linear, quadratic, and cubic terms), and recruiting center as fixed effects, and a kinship coefficient was used to adjust for family relatedness as a random effect. Single variant analyses and gene-based analyses were both conducted. For gene-based analyses, sequence kernel association test (SKAT), simple burden test, Madsen and Browning weighted burden test (MB), and variable threshold test (VT) were utilized for rare variants with MAF < 1% (Madsen and Browning, 2009; Price et al., 2010; Wu et al., 2011).

### Functional Validation

We also sought to explore the relationships between our top findings and other omic layers using the following GOLDN data: DNA methylation (measured with the Illumina Infinium 450K chip, *n* = 991) (Irvin et al., 2014) and gene expression (measured with RNA-Seq, *n* = 100 unrelated participants) as previously described (Sayols-Baixeras et al., 2016). The CpG sites of CD4+ T-cells within the genes containing significantly associated variants and the intergenic CpG sites near these genes were examined to test whether their methylation levels were associated with lipid levels or not. For transcriptional profiling, GOLDN participants were selected from the extremes of the BMI distribution. RNA was extracted from buffy coats using the TRIzol method (Thermo Fisher Scientific, Waltham, MA, United States) and the quality was evaluated using Bioanalyzer (Agilent Technologies, Santa Clara, CA, United States). We also fitted linear mixed models to test for associations of DNA methylation and gene-expression. We hypothesized CD4+ T-cells should reflect underlying epigenetic variation influencing blood lipids. Many key genes (e.g., *PPARs*) involved in lipid metabolism are expressed in lymphocytes and other immune cells (Chinetti et al., 2000; Bouwens et al., 2008). It was demonstrated that peripheral blood mononuclear cell gene expression profiles reflect nutrition-related metabolic changes. Responsive genes including *CPT1*, *ACAA2*, and *SCL25A20* were enriched for fatty acid metabolizing enzymes (Bouwens et al., 2007).

## Results

### Association Test Results

**FIGURE 1:**
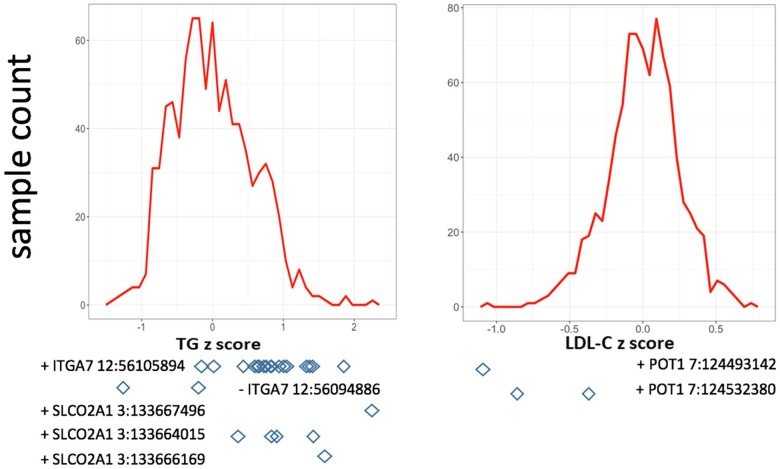
Distribution of residuals from the regression of phenotypes on their covariates. The diamonds indicate corresponding residual values of rare variant carriers. *X*-axis lists the *z*-score for residuals; *Y*-axis represents the sample counts.

In single variant tests, we found rs11171663 in *ITGA7* to be significantly associated with fasting TG levels (*P* = 7.66E-08), explaining approximately 3.2% of the total trait variance. The 16 participants carrying this variant exhibited significantly higher TG compared with non-carriers (mean of 260 mg/dL in carriers vs. 114 mg/dL in non-carriers, *P* < 0.05) (Figure 1 and Table 2). In the gene-based test, three genes were significantly associated with lipid traits, specifically *ITGA7* (TG, *P* = 1.77E-07), *SLCO2A1* (TG, *P* = 7.18E-07), and *POT1* (LDL-C, *P* = 3.00E-07). In the *ITGA7* gene-based test, the signal was driven by the same variant (rs11171663) identified in the single variant test. The severities of rare variant effects, predicted by PolyPhen2 (Ramensky et al., 2002) or SIFT (Ng and Henikoff, 2003), are listed in Table 2. The Bonferroni-corrected significance thresholds for single variant tests and gene-level tests were *P* < 5.98E-07 (0.05/83,577 rare variants) and *P* < 3.44E-06 (0.05/14,521 genes), respectively.

**Table 2:** Gene-based test results in GOLDN, augmented with corresponding single variant data.

Trait	Gene	Gene *P*-value	Method	Rare variant #	Rare variants with *P* < 0.10					
					Variant ID	Variant *P*-value	Variant effect direction	Rare allele carrier #	Variant effect	Allele frequency in ExAC
TG	*ITGA7*	1.77E-07	SKAT	13	12:56094886:G:T (rs375671999)	7.30E-02	-	2	T->K possibly damaging	4.5E-05
					12:56105894:G:A (rs11171663)	6.45E-08	+	16	T->I possibly damaging	0.01
	*SLCO2A1*	8.97E-07	MB	4	3:133664015:T:C (rs150345667)	5.76E-03	+	4	N->S benign	1.6E-03
		7.18E-07	VT		3:133666169:A:G (rs200472093)	1.11E-04	+	1	M->T benign	7.5E-05
					3:133667496:A:G (rs1415094638)	3.87E-03	+	1	L->P probably damaging	4.5E-04
LDL-C	*POT1*	1.32E-06	VT	5	7:124493142:C:G (rs1172142052)	1.30E-05	-	1	M->I tolerated	NA
		3.00E-07	MB		7:124532380:T:C^a^ (rs375440229)	1.73E-03	-	2	I->V benign	2.3E-04


*Rare variant #: the number of rare variant loci within the candidate genes; Rare allele carrier #: the number of samples who carry the rare variant; Variant P: *P*-value for the null hypothesis of the association test between the rare variant and its corresponding trait. ^*a*^The rare variant was carried by samples from the same family. ExAC: http://exac.broadinstitute.org/; SKAT, sequence kernel association test; MB, Madsen and Browning weighted burden test; VT, variable threshold test*.

### Replication Results

In the pooled HyperGEN and GENOA samples, *POT1*, *ITGA7*, and *SLCO2A1* approached significant associations (*P* = 0.020, *P* = 0.036 and 0.082, respectively) in the gene-based test (Supplementary Table 1). After Bonferroni correction, the significant threshold for association in this replication gene-based test is 0.05/3. Among the top single variants in these three genes in GOLDN, only one in *ITGA7* (rs11171663) and another one in *SLCO2A1* (rs150345667) were shared by HyperGEN and GENOA samples, but both associations failed to replicate (*P* > 0.05).

Our top TG findings were allowed to be tested for association with the corresponding trait in the HAPI Heart Study, which were recruited from the Amish community of Lancaster County, PA, who are descendants of about 200 original founding individuals. None of the *ITGA7* or *SLCO2A1* SNVs with *P* < 0.05 in GOLDN were found in HAPI Heart samples which are from a unique founder population. Variants in these two genes according to single variant test or these two genes according to gene-based test were not significantly associated with TG in HAPI Heart Study.

### Functional Validation

Transcripts of all the three top genes were observed from the buffy coats. The significant threshold for functional validation of gene-expression is 0.05/3 for multiple testing. In the gene transcript analysis, the expression levels of *SLCO2A1* and *ITGA7* approached a significant association with TG (*P* = 0.02 and *P* = 0.07, respectively), and *POT1* was not significantly associated with LDL-C (*P* > 0.1). The associations between the methylation status of CpG sites within or neighboring the three significant genes with lipid levels were not statistically significant after Bonferroni correction.

## Discussion

Genetic associations with fasting blood lipids have been extensively studied. Here, we sought to expand on that work by examining data from heterogeneous communities to leverage the population-specific nature of rare coding variants, striving for a more thorough understanding of the mechanisms underlying lipid metabolism.

In this study, we present preliminary evidence of rare variant determinants of circulating lipids. Three genes – *ITGA7*, *SLCO2A1*, and *POT1* – were significantly associated with fasting lipid levels in the discovery cohort. Two of these findings (*ITGA7* and *POT1*) were nominally replicated in the African American HyperGEN/GENOA sample but not in Old Order Amish, likely due to the genetic isolation of the latter. Additionally, the expression level of *SLCO2A1* was associated with fasting TG, suggesting regulatory relevance. Importantly, lack of replication in ethnically distinct cohorts supports the population-specific effect of rare variants on lipid traits, particularly because SNVs with *P* < 0.05 in the top three genes in GOLDN (the discovery cohort) were not found in HAPI Heart at all, and only few were shared with HyperGEN/GENOA.

The three genes harboring novel loci identified in our study have been implicated in lipid homeostasis. *SLCO2A1* is a lipid transporter protein, which could inhibit TG accumulation (Gimeno, 2007). *ITGA2*, an *ITGA7* homolog, was reported to be associated with coronary atherosclerosis in the Chinese Han population (Wang Y. et al., 2010), drawing attention to the ITGA gene family in the cardiovascular context. We also observed associations between *ITGA7* and LDL-C response to fenofibrate in another GOLDN study (Geng et al., 2018). In the conditional analysis after including LDL-C response to fenofibrate as a covariate, *ITGA7* is still significantly associated with fasting TG (*P* < 0.05). The associations between the top three genes and lipid levels were in part supported by our replication and functional assessments. The putative biological mechanism of POT1 needs further clarification.

In addition to identifying novel associations, we also tested variants within known lipid genes and corresponding traits. In GOLDN, variants within *APOE*, *PCSK9*, *LDLR*, and *APOB* were nominally associated with fasting LDL-C in single variant association tests (*P* < 0.05), and *PCSK9* and *LDLR* were nominally associated with fasting LDL-C in gene-based tests (*P* < 0.05). These findings are consistent with a previous study, which reported associations between LDL-C and *APOE*, *PCSK9*, *LDLR*, *APOB*, and *PNPLA5* (Lange et al., 2014). However, rare variants in *PNPLA5* (Lange et al., 2014) were not present in GOLDN and nor was the gene significantly associated with LDL-C, further highlighting the population-specific nature of some genetic determinants of lipids.

Our work also suggests that rare variants may play different roles in different populations. Heterogeneous effects of the same variant across different ethnic groups are due to differences in the genetic background or environment (Lin et al., 2007; Lu et al., 2017). Subjected to natural selection or genetic drift, inter-ancestry differences in the identification of rare coding variants across populations were observed as expected (Lu et al., 2017). In our study, rare variant rs11171663 in *ITGA7* was significantly associated with TG in Caucasian samples from GOLDN, but the direction of effect was opposite in African American samples from HyperGEN and GENOA, yet the variant still approached significance (*P* = 0.053). The hypothesis that this rare variant – or others identified in our study – may be positively or negatively associated with fasting lipid levels depending on the population merits attention in future studies in individuals of diverse ancestry. One limitation of our study is that we did not take the environmental factors for participants into account, which may interact with the variants and account for partial population difference as well.

## Conclusion

In this study, we presented preliminary evidence of novel rare variant determinants of fasting lipids, which may inform novel biomarkers of disease risk and treatment targets. We found statistically significant associations between *ITGA7* (*P* = 1.77E-07) and *SLCO2A1* (*P* = 7.18E-07) and triglycerides, as well as between *POT1* (*P* = 3.00E-07) and low-density lipoprotein cholesterol. Replication analyses yielded mixed results: in 3,183 African American samples from HyperGEN and GENOA study, the top genes achieved *P*-values of 0.04 (*ITGA7*), 0.08 (*SLCO2A1*), and 0.02 (*POT1*), but no associations approached significance in 770 samples from the HAPI Heart Study (*P* > 0.05), which implies exploration of genetic data from diverse population samples may enhance the identification of novel associations with rare variants. In GOLDN, the *P*-values for the association between gene transcript levels of *ITGA7* and *SLCO2A1* and fasting triglycerides were 0.07 and 0.02, respectively, highlighting functional relevance of our findings.

## Data Availability

The datasets generated for this study can be found in dbGaP https://www.ncbi.nlm.nih.gov/projects/gap/cgi-bin/study.cgi?study_id=phs000741.v2.p1. The other datasets used and/or analyzed during the current study are available from the corresponding author on reasonable request.

## Ethics Statement

Our study has been approved by the appropriate internal review boards at the University of Texas Health Science Center at Houston and all other participating institutions, and it abides by the Declaration of Helsinki principles.

## Author Contributions

MI, BH, SA, HT, JO, DA, and DZ designed the study. XG, VS, and DZ analyzed the data. TD, KR, and BM conducted the replication in HAPI heart study. XG, RS, BH, SA, and DZ wrote the manuscript. All authors participated in data interpretation, reviewed the manuscript, and approved the final version of the manuscript.

## Conflict of Interest Statement

The authors declare that the research was conducted in the absence of any commercial or financial relationships that could be construed as a potential conflict of interest.
