# Supplementary material for: An Exome-Wide Sequencing Study of the GOLDN Cohort Reveals Novel Associations of Coding Variants and Fasting Plasma Lipids
Source: Front Genet. 2019 Feb 26;10:158. doi: 10.3389/fgene.2019.00158 (PMC6399202; doi:10.3389/fgene.2019.00158)
Supplement: Supplementary file 1 [file Table_1.DOCX]

Supplemental Table 1. P values of gene-based tests for the associations between three genes and their corresponding traits in three cohorts

| TG |  |  |  |  |  |  |  |  |  |  |  |  |
| --- | --- | --- | --- | --- | --- | --- | --- | --- | --- | --- | --- | --- |
|  | HyperGEN & GENOA | |  |  | GOLDN |  |  |  | HAPI |  |  |  |
|  | Burden | MB | SKAT | VT | Burden | MB | SKAT | VT | Burden | MB | SKAT | VT |
| SLCO2A1 | 8.26E-02 | 1.23E-01 | 1.61E-01 | 2.50E-01 | 2.19E-04 | 8.97E-07 | 2.96E-03 | 7.18E-07 | 8.13E-01 | 8.94E-01 | 2.11E-01 | 5.48E-01 |
| ITGA7 | 3.67E-02 | 1.53E-01 | 1.22E-01 | 1.67E-01 | 2.47E-03 | 1.65E-01 | 1.77E-07 | 1.31E-02 | 3.35E-01 | 3.16E-01 | 4.54E-01 | 5.51E-01 |
| LDL-C |  |  |  |  |  |  |  |  |  |  |  |  |
|  | HyperGEN & GENOA |  |  |  | GOLDN |  |  |  | HAPI |  |  |  |
|  | Burden | MB | SKAT | VT | Burden | MB | SKAT | VT | NA |  |  |  |
| POT1 | 6.17E-01 | 5.01E-02 | 2.70E-01 | 2.00E-02 | 4.41E-04 | 1.05E-06 | 1.83E-02 | 1.32E-06 |  |  |  |  |
